# Non-invasive Brain Stimulation in Pediatric Migraine: A Perspective From Evidence in Adult Migraine

**DOI:** 10.3389/fneur.2019.00364

**Published:** 2019-04-12

**Authors:** Filippo Brighina, Vincenzo Raieli, Luca Maria Messina, Giuseppe Santangelo, Domenico Puma, Flavia Drago, Lucia Rocchitelli, Francesca Vanadia, Giuseppe Giglia, Salvatore Mangano

**Affiliations:** ^1^Dipartimento di Biomedicina, Neuroscienze and Diagnostica Avanzata (BiND), University of Palermo, Palermo, Italy; ^2^ARNAS Ospedali Civico Di Cristina Benfratelli, Palermo, Italy; ^3^Dipartimento di Promozione della Salute, Materno-Infantile, di Medicina Interna e Specialistica di Eccellenza “G. D'Alessandro”, University of Palermo, Palermo, Italy

**Keywords:** transcranial magnetic stimulation, transcranial direct current stimulation, non-invasive brain stimulation, pediatric migraine, therapeutics

## Abstract

Pediatric migraine remains still a challenge for the headache specialists as concerns both diagnostic and therapeutic aspects. The less ability of children to describe the exact features of their migraines and the lack of reliable biomarker for migraine contribute to complicate the diagnostic process. Therefore, there's need for new effective tools for supporting diagnostic and therapeutic approach in children with migraine. Recently, promising results have been obtained in adult headache by means of application of neurostimulation techniques both for investigating pathophysiological mechanisms and also for therapeutical applications. Non-invasive brain stimulation (NIBS) techniques like transcranial magnetic stimulation (TMS) and transcranial direct current stimulation (tDCS) indeed proved to be generally safe and showing also some evidence of efficacy particularly for the symptomatic treatment. On such basis, in the last years increasing interest is rising in scientific pediatric community to evaluate the potential of such approaches for treatment pediatric headaches, particularly in migraine, even if the evidence provided is still very poor. Here we present a perspective for application of TMS and tDCS technique in children migraine principally based on evidence coming by studies in adults.

## Introduction

Pediatric migraine remains a challenge for headache specialists, as concerns both diagnostic and therapeutic aspects. The low ability of children to describe the exact features of their migraines and the lack of reliable biomarkers complicate the diagnostic process, while symptomatic and prophylactic treatments are limited due to placebo effect and the parents' fear of pharmacological side effects (1–3). Therefore, there is a need for new effective tools for supplementing the existing diagnostic and therapeutic approaches in children with migraine.

Recently, promising results have been obtained in adult headache by applying neurostimulation techniques for investigation of pathophysiological mechanisms as well as for identification of potential clinical biomarkers, and last, but not least, of possible better-tolerated therapeutic alternatives (4). On such basis, over the last few years, the scientific pediatric community has become increasingly interested in evaluating these methods with respect to the therapeutic approach to pediatric headaches, particularly migraine.

Non-invasive brain stimulation (NIBS) techniques are defined as neurophysiological approaches for transcranial application of electrical currents or magnetic fields that are able to modulate brain activity, and are employed for investigating pathophysiology and also as diagnostic and therapeutic tools in many neuropsychiatric diseases (4). The first reported application of neurostimulation dates back to the first century AD, when Scribonius Largus relieved pain using the black torpedo, a bioelectric fish, delivering an electrical pulse to the painful area (5). Subsequently, from nineteenth century onward, new electrical generators were utilized; since then, the application of electric stimulation of the vagal nerve has been used, at first, for treatment of refractory epilepsy, and later, also in different pain states (4). Other NIBS techniques have also been experimented with for treatment of pain and other neuropsychiatric diseases in adults, but in the field of pediatric headache, only some anecdotal reports are available. The majority of these reports are principally aimed at exploration of safety issues associated with the techniques (6–9).

Neuromodulation can modify the activity of several brain networks by modulating neuronal excitability, and excitatory or inhibitory effects, depending on different stimulation parameters (polarity, duration, or frequency of stimuli) (4). The NIBS techniques have the relevant advantage of inducing brain changes by non-invasive stimulation, which does not require intervention for application of permanent leads, is painless and optimally tolerated, and can be employed in awake subjects at rest or during execution of different tasks. These techniques function through transcranial application of magnetic or electric currents (transcranial magnetic [TMS] and electrical stimulation [tES], respectively).

In TMS, weak but rapid electric currents are elicited in the brain regions through fast variation of magnetic field (4), which activates cortical neurons, triggering them to discharge; TMS can be delivered in a single pulse, double pulse, or trains of repeated pulses (repetitive TMS [rTMS]). The first modality has been principally employed to study brain physiology and for diagnosis of diseases of the motor system and pathways, but has also found therapeutic application in symptomatic treatment of migraine with aura attack (10).

Double-pulse TMS has found application in investigation of cortical facilitation and inhibition owing to the ability of paired stimulation to selectively modulate cortical inhibitory or facilitatory circuits depending on the interval between the pulses (11, 12). Further, rTMS can induce lasting effects determining prolonged neuroexcitability-related changes that remain beyond stimulation, suggesting the potential for therapeutic use in neuropsychiatric diseases with abnormal (increased or decreased) cortical excitability, especially for long-term treatment (13). Generally, high-frequency stimulation increases cortical excitability while low-frequency decreases it; however, several modifications of stimulation parameters allow flexibility in the brain responses obtained, depending further on different diseases (4, 9).

Conversely, tES functions through application of direct or alternating weak currents (0.5–2 mA), delivered via electrodes attached to the scalp. The initial, and yet most frequent, approach is based on application of direct currents (transcranial direct current stimulation [tDCS]); tDCS acts by modulating neuronal excitability. Contrary to TMS, tDCS is not able to induce direct neural activation but affects excitability through polarization. Anodic currents induce depolarization, increasing excitability and the probability of spontaneous firing, while an opposite inhibitory effect is induced by cathodal stimulation through neuronal hyperpolarization (13). Further, tDCS is able to induce long-lasting neuroplastic effects that have been found to be critically dependent on glutamate-NMDA neuro-transmission and represents the physiological basis for therapeutic application (4, 13).

Here we present a perspective about the potential of NBS techniques in children migraine based on data about safety, coming from studies on other disease in children, and on evidence about efficacy by TMS and tES studies in adult migraineurs.

## Safety of TMS and tDCS in the Pediatric Population

The safety of NIBS techniques has been mainly studied in the adult population, and there are only a few reports on their use in the pediatric population (6–9). These pediatric studies investigated mainly single-pulse or paired-pulse TMS protocols that are not of therapeutic interest. A recent report examined in detail the issue of safety of TMS and tDCS in children through an extensive review of the articles published till 2014; based on an electronic search, 48 studies were found and evaluated, including a population of more than 500 children, and adolescents aged 2.5–17.8 years (9). The NIBS methods were used in several disorders (autism, epilepsy, depression, etc.). In nine studies, patients underwent only a single stimulation session while in the others, designed for therapeutic purposes, more stimulation sessions were applied; the frequency and number of stimulation sessions varied across reports, ranging from repeated daily to weekly sessions. In these studies, TMS was the most commonly applied NIBS technique, with different parameters of stimulation on referred thresholds as control, globally reporting only 1.2% important negative side effects (seizure and syncope). Minor side effects were headache, scalp discomfort, fatigue, neck stiffness, etc. Headache is a more frequent side effect (11.5%), although it is temporary and usually does not need any therapy. Sixteen studies were found to have used tDCS, accounting for more than 190 subjects, and the methodology varied considerably for range of intensity, session duration, and session number. Serious side effects were not reported, while mild side effects (redness, tingling, itching sensation, etc.) were reported in cumulative analysis, with the frequency ranging from 1.5 to 11.5%; they were transitory and no medical treatments were needed. The authors' conclusion was that TMS and tDCS are safe (1% serious adverse effect); however, considering that the majority of the data obtained using these methods originate from adult studies, it is necessary to follow some precautions, such as not including subjects with alcohol consumption, epileptogenic medication intake, recent cranial trauma, or history of seizures. Further, the authors suggested searching for possible history of syncope in order to minimize the risks. The fact that headache was the more common mild side effect suggests the contraction of the muscles near the stimulation site as a possible cause. Headache, always mild and brief, was also reported in the sham groups (i.e., placebo stimulation), suggesting non-specific effects.

The tDCS appears to present fewer side effects, especially those related to the site of stimulation, and local symptoms are principally observed in the adult population, whereas no skin lesion is reported in children. In the adult population, repeated tDCS sessions did not appear to increase side effects (14); however, the lack of studies with prolonged repetition over time does not allow clear conclusions to be drawn regarding long-term safety of tDCS in these populations, even if studies on animal models suggest safety of long-term use (15).

Our actual conclusion on the safety of using TMS and tDCS in the pediatric population are limited by the low sample size (~500 subjects), variability in the stimulation parameters that does not allow correlation between specific parameters and side effects, few long-term studies, the fact that many studies are performed on other outcomes and not specifically to evaluate the safety via appropriate questionnaires or follow-up, the lack of correlation with structural, neurophysiological, and general data (MRI, different neurophysiological alterations, or blood test results).

## Diagnostic and Therapeutic Use of TMS and tDCS in the Pediatric Migrainous Population

Several cortical and subcortical areas are involved in the pathogenesis of pain and migraine; a central role is played by the trigeminocervical complex, which has sensitive afferents and connections with the autonomic nervous system, as well as other subcortical and cortical centers. The trigemino-vascular system and trigemino-autonomic reflexes are believed to be involved in the main mechanism of migraine pain through multiple vasoactive peptides (calcitonin gene-related peptide, substance P, vasoactive intestinal peptide, pituitary adenylate cyclase-activating peptide, etc.) (16). Cortical and subcortical areas (the occipital and associative cortices, hypothalamus, periaqueductal gray, and locus coeruleus) are believed to activate, inhibit, or modulate the trigeminocervical complex (17). Peripheral and central sensitization mechanisms are invoked as causes of signs and symptoms of migraine attack and chronicization (18). On these bases, it is reasonable that each of the aforementioned nodes could represent a target of putative non-pharmacological strategies.

Visual aura has been extensively investigated as a marker of cortical dysfunction. The TMS has been used to analyze cortical excitability through the phosphene thresholds in migraineurs and controls (19–21). A single pulse applied to the visual cortex can induce an artificial percept or “phosphene,” which may be enhanced by adding a conditioning stimulus. The evoked phosphenes increase depending on the stimulation intensity, allowing establishment of the “phosphene threshold” of a subject. The phosphene threshold may also be modulated by TMS stimuli applied to the associated cortical area. Phosphene-induction using TMS allows assessment of the occipital cortex excitability in subjects with and without migraine. In the adult population, data suggest the existence of primary visual cortex hyperexcitability, especially in migraine with aura (21). These studies are limited by variability of stimulation parameters in absence of uniformly adopted protocol for measuring phosphenes; however, response to TMS seems to be a very promising biomarker for migraine. Recently, anodal tDCS application to the temporal pole has been shown to enhance interictal excitability of the visual cortex in migraineurs, restoring normal habituations, underlining the role of the temporal pole in visual processing (22).

Evidently, in the pediatric population, the data are few and sparse, and show lower phosphene thresholds in interictal migraineurs vs. controls, with changing excitability levels 1–2 days before migraine attacks, reflecting the relation of fluctuating excitability to the migraine cycle (23). To date, to our knowledge, only one pivotal study (24) in adolescents affected by migraine has explored the therapeutic use of rTMS as a preventive treatment, showing reduction in the number of headache days, use of abortive drugs, and MIgraine Disability Assessment score, and safe use and few side effects. However, the study has several limitations, such as an open-label design and small sample population.

The matter, however, is worth investigating further because NIBS showed promise in the treatment of pain and migraine in adults. High-frequency magnetic stimulation of the motor cortex indeed showed level A evidence of effectiveness against neuropathic pain (13). A large randomized study on a population migraineurs with visual aura using single-pulse stimulation for acute attacks showed significantly greater improvement following real stimulation, compared with sham stimulation, at 2 and 24 h with regard to the following outcome measures: pain relief, nausea, and phono- and photo-phobia, in the absence of side effects (10). The limitations of the study were mainly the sample population exhibiting only migraine with visual aura; moderate gain on sham effect (17%), lower than that reported using traditional therapeutic drugs such as triptans (25); and the difficulty in achieving a true blind effect with this method. The results from the ESPOUSE Study (26) (observational post-marketing study) support the possible therapeutic effect of TMS as a preventive agent against adult migraine, with low-to-mild side effects and no serious adverse effects. Recently, the US Food and Drug Administration has authorized the use of single-pulse TMS for abortive therapeutic purposes (27).

In chronic migraine, the available results on rTMS prophylactic therapy are contradictory, with the few published studies having small sample populations, lack of consensus regarding brain targets, variation in stimulation parameters, and issues related to the utilized masks, causing difficulties in their comparison and establishment of clear conclusions regarding the effectiveness of TMS against chronic migraine (28–30). However, the generally reported lack of side effects and the potential of this method make its use promising in the pediatric population, where the parents' fear of side effects is an important limitation on the use of pharmacological drugs (31).

## tDCS in Pain and Migraine

To our knowledge, studies using tDCS in the treatment of pediatric migraine and pain have not yet been published. However, observing the increasing number of instances of tDCS use in adult pain and considering the data from its use in other pediatric disorders, we can hypothesize the effective application of this technique in pediatric pain. Evidence regarding the effect of tDCS on adult patients with migraine is still inconclusive; however, two studies applying cathodal currents over the primary visual cortex showed a significant amelioration of the symptoms compared with the baseline, pretreatment condition with respect to duration, intensity, and severity of attacks, even though only the intensity changed significantly compared with placebo sham stimulation (32, 33). No severe adverse effects were reported, with good tolerability. In a meta-analysis, Luedtke et al. (34) concluded that clinical data does not support the use of tDCS in the treatment of pain and migraine. However, the authors advise designing studies with larger sample populations using shared protocols on stimulation parameters and stimulation sites to better evaluate the effectiveness of this method, which is promising due to its low cost, easy applicability, non-invasiveness, and lack of serious adverse effects. These aspects are even more relevant in the context of the pediatric population, where tolerability, and non-invasiveness are critical characteristics for its consideration for therapeutic treatment.

## Potential Strategies for TMS and tDCS Application in Pediatric Pain and Migraine, and Conclusions

The Cochrane reviews do not provide clear conclusions regarding the effectiveness of TMS and tDCS against adult chronic pain, although small benefits appear to have been observed. However, the authors point out many biases and important heterogeneities of these studies (30).

At the moment, it is not possible to establish useful guidelines on the use of TMS and tDCS in the treatment of pediatric migraine and, in general, for pediatric pain treatment. However, adult studies as well as preliminary pediatric reports show that the application of these techniques is safe, with few side effects, potentially low costs, and easy applicability. Furthermore, in adults, for some serious painful disorders, such as chronic regional pain syndrome, level A evidence has been obtained regarding the pain-relieving effects of these techniques. Preliminary reports, principally in adults but also in the pediatric population, suggest that migraine may represent an effective therapeutic target. Moreover, NBS of cortical areas as DLPFC, that has been explored in migraine, was found to be effective for treatment of other conditions, that are comorbid with disease, sharing also a stimulation target employed for migraine treatment, like DLPFC. Among these disorders, in addition to the role played by the psychiatric diseases, of particular importance is obesity which also favors the chronification of migraine (35, 36). Due to the large prevalence of the disease and the disability associated with it, and also considering the parents' relevant fears and concerns regarding pharmacological therapies, especially for continuing preventive treatment, pediatric migraine appears to be an optimal candidate for future studies on therapeutic NIBS. Therefore, this topic is worth exploring further through rigorous, opportunely suited randomized controlled trials with uniform diagnostic protocols, and stimulation parameters to reveal the real therapeutic potential of NIBS techniques against pediatric migraine.

## Author Contributions

FB and VR study conception. LM, GS, DP, FD, and LR data collection. FB, VR, FV, and GG data analysis. FB, VR, and SM manuscript writing. FB, VR, SM, FV, and GG manuscript revision.

### Conflict of Interest Statement

The authors declare that the research was conducted in the absence of any commercial or financial relationships that could be construed as a potential conflict of interest.
